# Novel 360° lumbar arthroplasty: Surgical technique and procedural details

**DOI:** 10.1016/j.xnsj.2026.100863

**Published:** 2026-02-05

**Authors:** Jared D. Ament, Jack Petros, Cooper Gardner, Amir Vokshoor

**Affiliations:** a Neurosurgery & Spine Group, 2901 Wilshire Blvd., Suite 105, Santa Monica, CA 90403, United States; b Institute of Neuro Innovation, Santa Monica, CA, United States; c Cedars Sinai Medical Center, Los Angeles, CA, United States; d Saint John’s Health Center, Santa Monica, CA, United States

**Keywords:** Artificial disc replacement, Artificial facet replacement, Dynamic posterior stabilization, Motion preservation, 360° arthroplasty

## Abstract

**Objective:**

To describe the operative technique, dedicated intraoperative calibration workflow, and perioperative management protocol for 360° lumbar arthroplasty, a dual-approach, motion-preserving alternative to fusion addressing concurrent disc and facet pathology at a single lumbar level.

**Background:**

Conventional fusion restricts segmental motion, potentially increasing stress on adjacent levels. The combination of the Prodisc L, artificial disc replacement, and the Total Posterior System from enables circumferential reconstruction of the lumbar motion segment while preserving physiologic kinematics. A novel intraoperative calibration device allows precise alignment of the Total Posterior System to the artificial disc replacement, optimizing segmental center of rotation and motion preservation.

**Methods:**

Patient selection, preoperative imaging, sequential anterior-posterior surgical technique, dedicated intraoperative center of rotation calibration protocol, device sizing, and postoperative rehabilitation protocols are detailed. Biomechanical validation of the calibration device is incorporated to support its clinical application.

**Results:**

Early results of 24 patients over 2 years are encouraging. Total VAS and ODI scores decreased by 9 (89%) and 50 (88%) points, respectively. The SF36 PCS/MCS scores increased by 63/49 (114%/100%). A total of 9 patients were discharged the same day, while 14 were sent to a post-op rehab center for 1 to 3 days. Average return to light physical activity (swimming, golfing, fencing, hiking, gym) was 4 weeks (SD2.2).

**Conclusions:**

This 360° motion-preserving construct integrates validated anterior and posterior arthroplasty systems with a novel intraoperative calibration to restore both disc and facet function, representing an evolution in lumbar spine surgery.

## Introduction

Lumbar degenerative disease often presents with combined disc space and facet disease, leading to axial pain, neural compression, and possible instability at the affected segment [1]. Traditional surgical management for symptomatic lumbar degeneration has largely centered on neural decompression supplemented by instrumented fusion when mechanical instability or structural collapse is present. While fusion reliably addresses pain generators arising from instability or stenosis, it does so at the cost of eliminating segmental motion. The resultant increase in mechanical stress at adjacent levels contributing to the well-documented phenomenon of adjacent segment disease has thus motivated exploration of motion-preserving alternatives [[2], [3], [4], [5]].

Artificial disc replacement (ADR) aims to address these drawbacks by restoring disc height and preserving mobility. Clinical outcomes have consistently demonstrated lower reoperation rates, less adjacent-segment degeneration, and overall superior outcomes compared with fusion in appropriately selected patients [3,4]. However, even with contemporary devices/options, ADR alone cannot (and should not be used to) fully address facet arthropathy, severe stenosis due to facet disease and/or ligamentous hypertrophy, or spondylolisthesis. Conversely, posterior dynamic stabilization systems address pure posterior pathology, modulating facet loading, reducing abnormal shear, and preserving motion while relying on an intact disc space.

The 360° lumbar arthroplasty (*methods patent pending*) is a novel motion-preserving approach that has arisen from these complementary but individually incomplete strategies, combining ADR with Total Posterior System (TOPS) to restore anterior disc height/mechanics, allow for comprehensive decompression of the neural elements, and provide posterior facet replacement and stability [[6], [7], [8], [9], [10], [11]]. This dual-implant strategy provides circumferential reconstruction, decompression, dynamic stabilization, and motion preservation in a single lumbar segment. In this article, we provide a comprehensive description of the 360° lumbar arthroplasty surgical procedure. We detail how the sequential integration of ADR and TOPS synergistically restores both disc and facet biomechanics, ultimately preserving the kinematics of the lumbar segment.

## Patient selection and preoperative evaluation

360° lumbar arthroplasty is indicated in skeletally mature patients who have failed at least 3 months of conservative therapy for treatment of leg pain with or without back pain at one vertebral level between L2 and S1 stemming from (1) degenerative spondylolisthesis or retrolisthesis up to Grade III and (2) moderate to severe lumbar spinal stenosis and (3) thickening of the ligamentum flavum or scarring facet joint capsule and (4) lumbar degenerative disc disease (DDD). The DDD is further defined as “severe” and/or asymmetric.

Patient eligibility is determined according to IRB-approved inclusion/exclusion criteria as illustrated in Table 1.

**Table 1:** Study inclusion and exclusion criteria.

Category	Criteria
Inclusion criteria	Moderate to severe leg pain and disability due to lower back pain
	Neurogenic claudication
	Pathology at the level to be treated (L2/3, L3/4, L4/5, or L5/S1) demonstrating all of the following: • Degenerative spondylolisthesis or retrolisthesis up to Grade III as determined by flexion/extension radiographs • At least moderate lumbar spinal stenosis • Thickening of the ligamentum flavum and/or scarring of the facet joint capsule identified on MRI or CT
	Degenerative disc disease (DDD) at the affected level between L2 and S1 with radiographic confirmation of one or more of the following: • Instability (≥3 mm translation or ≥5° angulation) • Decreased disc height >70% (or <6 mm remaining height) • Asymmetric disc degeneration on coronal imaging • Scarring or thickening of the annulus fibrosus • Herniated nucleus pulposus • Vacuum phenomenon
Exclusion criteria	Spondylolisthesis greater than Grade III
	Traumatic or dysplastic spondylolisthesis
	Back pain or nonradicular leg pain of unknown etiology
	Known allergy or sensitivity to nickel, PEEK, titanium, cobalt chrome, polyurethane, polyethylene, chromium, or molybdenum
	Clinically compromised vertebral bodies at the affected level due to traumatic, neoplastic, metabolic, or infectious pathology
	Scoliosis >20° by major Cobb angle
	Vertebral endplates smaller than 34.5 mm (medial–lateral) or 27 mm (anterior–posterior)
	Morbid obesity (BMI >40 kg/m²)
	Osteoporosis (T-score<−2.5), unless treated for ≥3 mo with denosumab (Prolia), teriparatide (Forteo), or abaloparatide (Tymlos)
	Paget’s disease, gout, osteomalacia, osteogenesis imperfecta, thyroid or parathyroid disorders, or other metabolic bone disease not stabilized with medication for ≥1 y
	Active infection (systemic or local)
	Active hepatitis
	AIDS, HIV infection, rheumatoid arthritis, or other autoimmune diseases
	Tuberculosis (active or within the past 3 y)
	Active malignancy or history of invasive malignancy (excluding nonmelanoma skin cancer) without disease-free status for ≥5 y
	Any condition requiring medications known to interfere with bone or soft tissue healing, or receiving radiation therapy during the study period
	Cauda equina syndrome, or neurogenic bowel or bladder dysfunction
	Vascular claudication due to severe arterial insufficiency
	Sustained pathologic lumbar fractures or multiple vertebral or hip fractures
	Significant peripheral neuropathy in a stocking-like distribution
	Insulin-dependent diabetes mellitus not well controlled (HbA1c <7%)
	Immunosuppression or systemic steroid use for >1 mo within the past year
	Current anticoagulant use (other than aspirin), unless safely discontinued prior to surgery
	Life expectancy <3 y
	Major mental illness or symptoms of psychological origin
	History of chemical or alcohol dependency or abuse within the past year
	Smoking >1 pack of cigarettes per week or frequent use of chewing tobacco
	Pregnancy
	Active spinal litigation
	Active workers’ compensation claim
	Current incarceration
	Participation in another investigational study within 30 d prior to surgery

## Surgical steps

### Overview

**Fig. 1:**
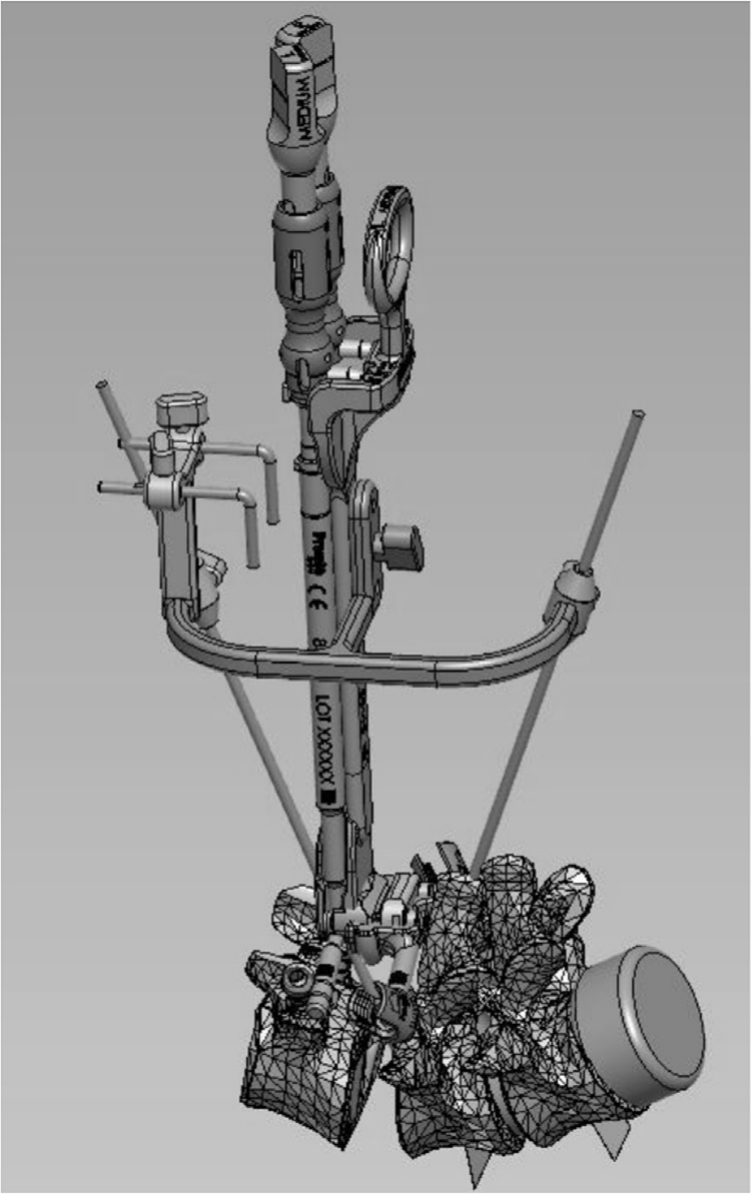
Custom intraoperative calibration device used to align the Total Posterior Spine System (TOPS) device relative to the anterior artificial disc replacement (ADR) to identify and match the segmental center of rotation (COR).

The procedure follows an intentionally sequential anterior-posterior approach. The anterior Prodisc L restores disc height, segmental alignment, and sagittal balance, while the posterior TOPS system allows for thorough decompression, resection of degenerated facets, and stabilization of the motion segment after the facetectomy. This sequence ensures accurate restoration of the anterior column prior to sizing the TOPS posteriorly. This is critical given the pedicular diameter sizing needed for the TOPS. Optimal disc height is first established using trial implants, achieving physiologic tension without overdistraction, preferably mirroring normal adjacent levels. During the posterior sizing and trialing process, a custom intraoperative calibration device (Fig. 1) is utilized to guide TOPS placement. Note, this is not part of the normal TOPS procedure in isolation. This calibration step ensures adequate physiologic kinematics and motion around the ADR’s segmental center of rotation (COR), guiding precise posterior placement of the TOPS device. Anchoring screw depths are adjusted as needed during this step. The TOPS device is then sized for length and pedicle distance and secured in place in the standard fashion.

### Anterior phase—Prodisc L total disc replacement

The patient is first positioned supine and neutral on a radiolucent table to enable unobstructed circumferential fluoroscopy. The operative level is localized with a lateral C-arm view and a straight metal marker to guide incision planning. The vascular cosurgeon makes a standard mini-open retroperitoneal approach is performed through a transverse incision, mobilizing the rectus muscle and developing the retroperitoneal plane. Vascular mobilization depends on level: at L3 to L4 and L4 to L5, the left iliac vessels are mobilized rightward; at L5 to S1, the common iliac vessels are retracted laterally and superiorly, often requiring ligation of the middle sacral vessels.

With the disc space exposed, midline alignment is confirmed fluoroscopically and marked on the adjacent vertebral bodies. A complete discectomy is performed with meticulous clearance of all disc material and posterolateral corners. The segment is remobilized, and disc height restored using a Vertebral Body Spreader to the posterior margin while preserving endplate integrity.

**Fig. 2:**
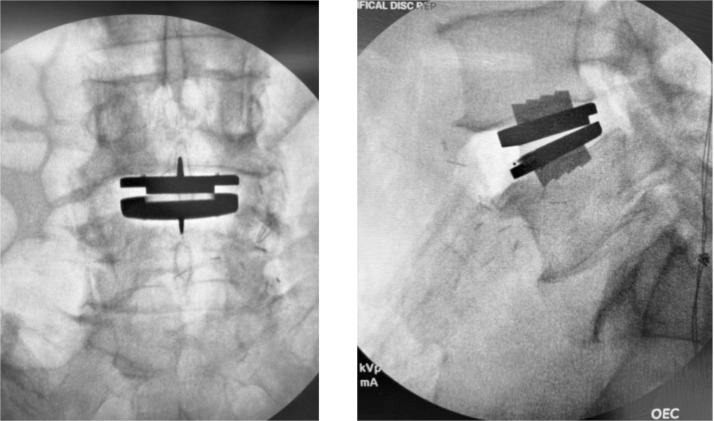
Anteroposterior (AP) and Lateral intraoperative fluoroscopic radiographs following completion of the anterior phase total disc replacement implantation, demonstrating final positioning of the Prodisc L artificial disc replacement implant.

Trial implants are inserted to determine optimal footprint, lordotic angle, and height, with the goal of maximizing endplate coverage to reduce the risk of subsidence. Once finalized, the chisel is guided along the trial to create keel tracks. The definitive Prodisc L endplates are then inserted in a collapsed position, aligned with chisel cuts, and malleted to the posterior vertebral margin. Experience has taught us that posterior placement (posterior marginal line) is most ideal for this particular construct. This ensures that the TOPS positioning can achieve adequate biomechanical coupling with the natural COR of the ADR. Following distraction, the ultrahigh molecular weight polyethylene inlay is inserted and locked into the inferior endplate. Proper seating is confirmed by verifying “NO STEP” and “NO GAP” at the anterior edge. Final implant positioning is verified with anteroposterior (AP) and lateral fluoroscopy before layered closure of the anterior wound (Fig. 2). The patient is then repositioned prone for the posterior phase.

### Posterior phase—Premia TOPS system

The patient is repositioned prone on a flattened Wilson Frame. A Jackson OSI table had been previously recommended, but hyperextension was noted prior to TOPS placement, and this was felt to be less than ideal for final placement/positioning and alignment. Once prone, a midline incision exposes the lamina and facet complex. Four polyaxial pedicle screws are placed bilaterally under fluoroscopic guidance, ensuring symmetrical interpedicular spacing and tulip height alignment. A proprietary pendulum guide assists in achieving proper lateral-medial trajectory while maintaining a minimum 6 mm gap between adjacent tulip heads. It is critical ensure that screw positioning will allow the TOPS device to sit in-line and perpendicular to the disc plane. It is also important to not violate the superior facet capsule. After screw placement, a wide decompression is performed, including a full laminectomy and bilateral facetectomies at the index level, extending laterally to the pars interarticularis for full exiting and traversing nerve root decompression. Adequate resection of the superior articular process is required to ensure a pedicle-to-pedicle opening that allows for motion without neural impingement. Adjacent spinous processes are often partially shaved (to fit the device) but mostly preserved. Further reduction of any remaining spondylolisthesis ought to be avoided.

**Fig. 3:**
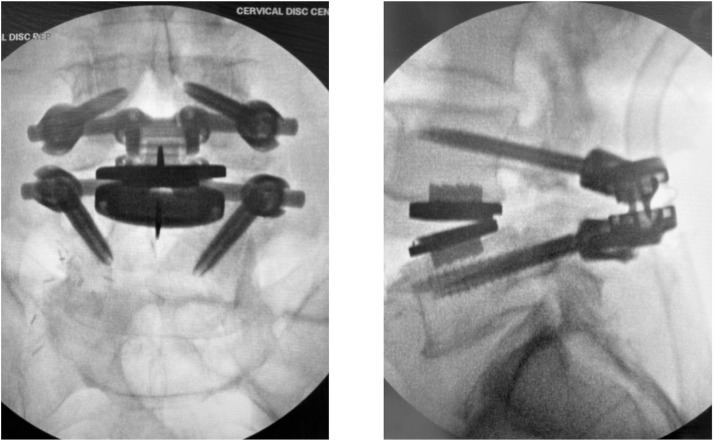
Anteroposterior (AP) and Lateral intraoperative radiographs after posterior phase Total Posterior Spine System (TOPS) implantation, forming the completed 360° motion-preserving construct.

**Fig. 4:**
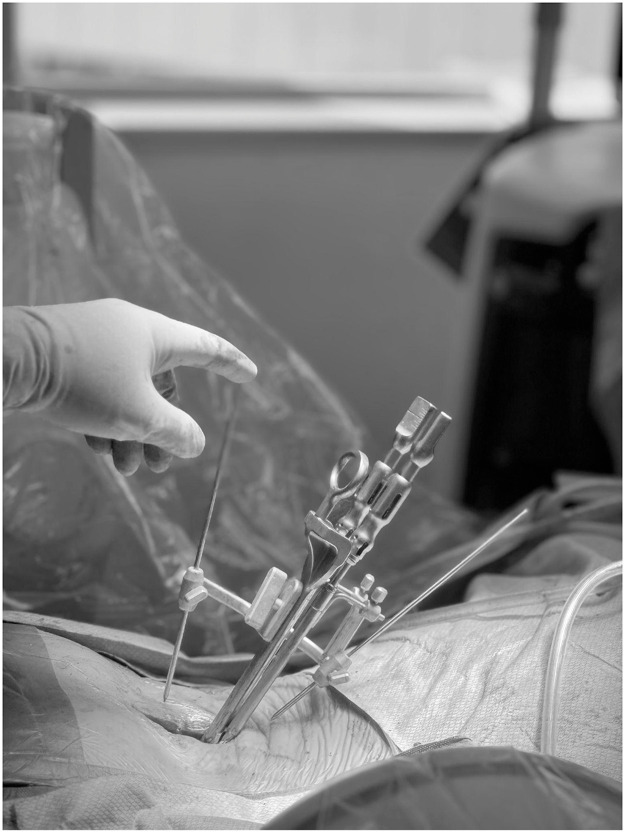
Clinical photograph exhibiting the custom calibration device being utilized intraoperatively to guide the alignment of the Total Posterior Spine System (TOPS) device relative to the artificial disc replacement (ADR).

Prior to insertion, the TOPS device is filled with saline, and screw polyaxiality is unlocked. A custom intraoperative calibration device is then attached to identify the appropriate TDR COR as it relates to the TOPS positioning (Fig. 4). Note, this is not a standard step for the standalone TOPS procedure and is described further in the following section. The appropriately sized implant, oriented cranially (“UP”), is inserted using the TOPS Inserter to maintain alignment, avoiding forceful seating. The Inserter must remain attached until all four set screws are secured, locking the construct to allow physiologic motion while restricting abnormal translation. Final fluoroscopy confirms balanced motion, appropriate COR alignment, and optimal sagittal positioning before layered closure (Fig. 3).

### Calibration device

Following ADR placement and prior to posterior instrumentation, a specialized intraoperative calibration device is utilized to determine the true segmental COR. This is highly dependent on ADR and TOPS positioning, in isolation and in relation to each other. This device enables real-time dynamic fluoroscopic assessment of segmental mobility and alignment at the operative level (Fig. 4). Incongruency or mismatch (malposition of the TOPS resulting in a COR that is nonphysiologic with the kinematics of the ADR) can caused segmental and possibly adjacent segment kinematic issues.

**Fig. 5:**
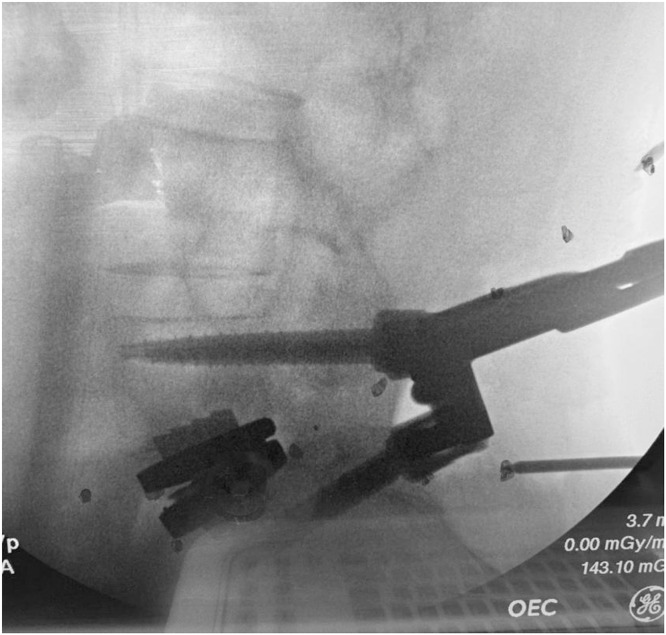
Fluoroscopic mapping of the segmental center of rotation (COR) using the calibration device to determine the optimal sagittal positioning of the Total Posterior Spine System (TOPS).

The calibration protocol begins by mounting the tool (modified trial) to the pedicle screws with set screws, like the standard trialing step for the TOPS procedure (Fig. 4). Positioning of this device requires precise AP and lateral fluoroscopic orientation prior to “advancing the pins.” The two small parallel L-shaped pins must be midline to the spine for final orientation on AP fluoroscopy. On lateral views, fluoroscopy angles must be adjusted to ensure the two L-pins are perfectly aligned (without parallax), horizontally and vertically. Once the correct orientation is achieved, the fluoroscopy machine is lowered to the level of the spine only. No rotation/wag or other adjustments should be made at this point. The long straight pins are then advanced as close to the patient as possible, ensuring that the at least the tips of the pins, the TOPS, and the ADR are all visualized in a single fluoroscopy lateral X-ray. Using a ruler or straight edge, the anterior and posterior long pin trajectories are mapped out on the fluoroscopy screen toward the ADR (Fig. 5). The point of intersection correlates with the COR of the ADR. At this point, the TOPS device (via the pedicle screws) can be adjusted ventrally or dorsally as needed for the COR to be in the correct anatomic position. This workflow is reproducible, takes less than 5 minutes once mastered, and ensures alignment symmetry to prevent shearing forces or motion conflict between the anterior and posterior implants.

### Biomechanical validation of the calibration device

**Fig. 6:**
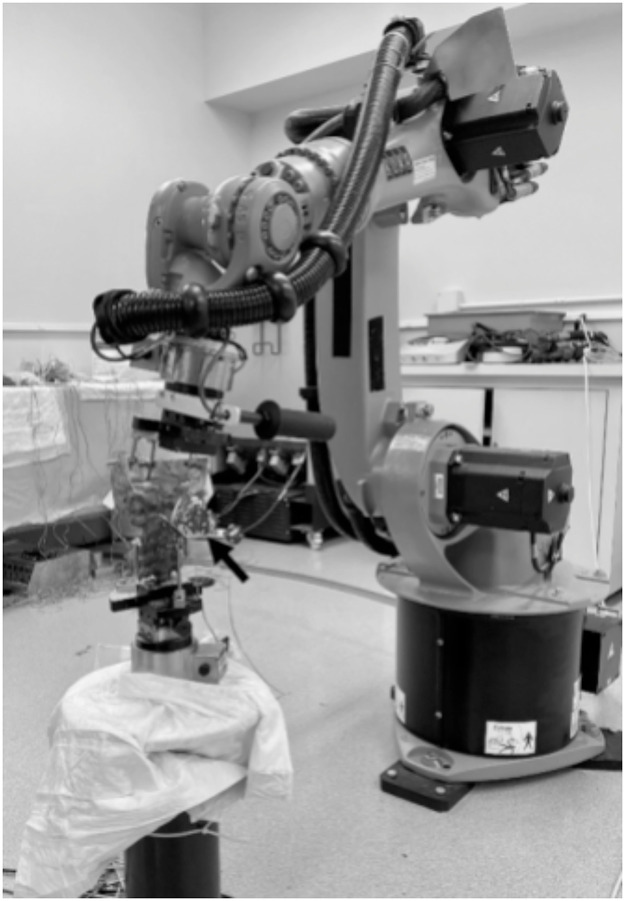
Example setup of the robotic testing system for biomechanical validation of the 360° lumbar arthroplasty construct.

We are currently calibrating with the Cleveland Clinic to biomechanically validate both the calibration tool and 360-construct through cadaveric robotic testing (Fig. 6). Lumbar spines (L3–S1) were instrumented with motion-tracking markers and tested using a 6° of-freedom robotic platform (Kuka KR16 with simVITRO software) under pure moment loading in flexion–extension, lateral bending, and axial rotation. Eight conditions were tested, including a native spine, a TDR+TOPS construct with COR alignment, and COR misalignments of 2 mm to 10 mm in the sagittal plane. Testing is being performed according to precise loading protocols (±7.5 Nm) with strict termination criteria (<0.05°/s rotation rates and torque/force thresholds) to ensure reproducibility. After each intervention, the specimen was returned to the robot for re-evaluation. Kinematic data collected at L3 to L4, L4 to L5, and L5 to S1 revealed that properly aligned constructs closely replicated native motion, whereas increasing COR misalignment induced abnormal segmental motion patterns.

Early results suggest that the intraoperative calibration tool plays a critical role in achieving COR precision and maintaining physiologic kinematics during the 360° arthroplasty procedure. Repeat testing is being conducted to confirm the reliability of the results.

## Postoperative management

Standard closure techniques are used, and a posterior Hemovac drain is always left subfascially. Patients are typically managed in an outpatient setting, ambulating within hours of discharge without a brace. Walking is encouraged immediately, with heavy lifting limited to <10 lbs for 1 week, followed by a gradual return to full activity by 6 to 12 weeks. Physical therapy begins within 1 to 2 weeks, focusing on motion, core activation, gait training, and progressive lumbar stabilization. This protocol directly contradicts the conventional post-op fusion management mantra of no “BLT” (bending, lifting, twisting). Follow-up imaging includes standing AP/lateral, flexion–extension, and lateral bending X-rays at 6 weeks, 3 months, 6 months, 12 months, and annually thereafter.

## Clinical pearls

During ADR trialing, the shortest posterior height implant should be used that best matches the patient’s native anatomy, limiting post-op distraction radiculitis. The ADR should be positioned midline and as posteriorly as possible. This will ensure easier COR calibration when the TOPS is positioned posteriorly. Similarly, the TOPS device’s final position ought to be as ventral as possible to ensure predictable and intended motion kinematics of both devices. Standard TOPS placement principles otherwise still apply. The pedicle screw tulip heads should be aligned under fluoroscopy in the lateral and coronal planes to ensure symmetric load bearing and prevent rotational imbalance. Calibration of the TOPS at each phase is critical, ensuring midline placement with respect to the pedicles and the ADR. Sizing of the TOPS should similarly not distract the segment but find a natural, anatomic position. The vertical alignment of the final TOPS position should be perpendicular to the disc space/ADR, and fluoroscopic verification of the two posterior vertical lines being parallel will limit excessive device wear.

## Discussion

The combination of Prodisc L ADR and Premia TOPS system offers a novel 360° motion-preserving solution for concurrent lumbar disc and facet pathology [6]. Surgical treatment of symptomatic lumbar spondylolisthesis with advanced DDD has traditionally centered on neural decompression with instrumented fusion for stabilization [12,13]. Although fusion provides reliable stabilization and pain relief, it does so by abolishing natural segmental motion. The long-term benefits vary, and the loss of motion has been shown to increase biomechanical load on adjacent levels. This predisposed patients to adjacent segment disease and additional surgery [14,15]. Furthermore, the authors contend that fusion is fundamentally antiquated, nonphysiologic, and has become convention simply because alternative technologies/methodologies have been unavailable.

This novel surgical procedure is performed through sequential anterior and posterior approaches. The anterior phase restores disc height and alignment via an open retroperitoneal discectomy and ADR implantation, while the posterior phase achieves direct central and neuroforaminal decompression via laminectomy and facetectomies with stabilization (and motion preservation) with the TOPS insertion. A custom intraoperative calibration device ensures precise alignment, achieving approximate near-native lumbar biomechanics.

Several limitations warrant consideration. Current clinical observations remain preliminary, with limited sample size and follow-up. While early cadaveric data support COR-matched kinematics, in vivo biomechanical behavior may differ due to muscle forces, healing response, implant settling, and patient-specific variation. The 360° lumbar arthroplasty combining Prodisc L and TOPS has been performed by a small number of surgeons in the United States to date, reflecting the procedure’s early adoption phase. Our center has an ongoing formal, IRB-approved, clinical trial that, at this time as enrolled 24 patients with up to 2.5 years of follow-up. Additionally, TOPS is a relatively recent FDA-approved motion preservation device, and broader surgeon familiarity and procedural standardization are still evolving. At present, only a single intraoperative COR calibration instrument of this design is available, which further limits generalizability and underscores the need to evaluate reproducibility across surgeons and institutions. Additionally, while ADR has been routinely performed at multiple levels, the TOPS device was only designed for use at a single vertebral segment. This is a notable limitation, seemingly preventing the use of this methodology at more than a single lumbar level. It is however, noteworthy that several surgeons have already started investigating multilevel TOPS devices, utilizing a dual-headed screw technique across the shared middle pedicle. This is, of course experimental on its own accord with unclear load-sharing biomechanics. While there may be future applications for multilevel 360-lumbar arthroplasty, the authors have not included this in the methodology or as part of the initial trial.

Future investigations, including larger cohorts, longer-term follow-up, comparative control groups, and dynamic functional motion analytics, are required to define clinical durability, alignment tolerances, and how this all relates to patient-reported outcomes.

## Conclusion

Early results suggest that the 360° lumbar arthroplasty represents a reproducible, motion-preserving alternative for patients with combined disc and facet degeneration. The integration of a novel intraoperative calibration device ensures precise alignment of the posterior TOPS system relative to the anterior TDR, optimizing segmental kinematics. This dual-implant construct preserves physiologic motion while providing circumferential reconstruction, supporting its use in appropriately selected patients.

## Patient Informed Consent Statement

All patients signed an expanded trial informed consent that acknowledged that they were entering into an investigational trial.

## Declarations of competing interests

The authors declare that they have no known competing financial interests or personal relationships that could have appeared to influence the work reported in this article.
