# Corrigendum to “Novel 360° lumbar arthroplasty: Surgical technique and procedural details” [North American Spine Society Journal (NASSJ) 25 (2026) 100863]

**DOI:** 10.1016/j.xnsj.2026.100946

**Published:** 2026-10-03

**Authors:** Jared D. Ament, Jack Petros, Cooper Gardner, Amir Vokshoor

**Affiliations:** a Neurosurgery & Spine Group, 2901 Wilshire Blvd., Suite 105, Santa Monica, CA 90403, United States; b Institute of Neuro Innovation, Santa Monica, CA, United States; c Cedars Sinai Medical Center, Los Angeles, CA, United States; d Saint John’s Health Center, Santa Monica, CA, United States

The authors regret that they prematurely included a paragraph and explanation around the biomechanics testing that was preliminary and no longer current/accurate. Multiple iterations and updates to the biomechanics testing, protocol, and methodology have since been made so we ask that the following section be disregarded:

*Biomechanical validation of the calibration device*

We are currently calibrating with the Cleveland Clinic to biomechanically validate both the calibration tool and 360-construct through cadaveric robotic testing (Fig. 6). Lumbar spines (L3–S1) were instrumented with motion-tracking markers and tested using a 6° of-freedom robotic platform (Kuka KR16 with simVITRO software) under pure moment loading in flexion–extension, lateral bending, and axial rotation. Eight conditions were tested, including a native spine, a TDR + TOPS construct with COR alignment, and COR misalignments of 2 mm to 10 mm in the sagittal plane. Testing is being performed according to precise loading protocols (± 7.5 Nm) with strict termination criteria (< 0.05°/s rotation rates and torque/force thresholds) to ensure reproducibility. After each intervention, the specimen was returned to the robot for re- evaluation. Kinematic data collected at L3 to L4, L4 to L5, and L5 to S1 revealed that properly aligned constructs closely replicated native motion, whereas increasing COR misalignment induced abnormal segmental motion patterns. Early results suggest that the intraoperative calibration tool plays a critical role in achieving COR precision and maintaining physiologic kinematics during the 360° arthroplasty procedure. Repeat testing is being conducted to confirm the reliability of the results.

And the following section considered:

*Biomechanical Validation of the Calibration Device*

Current biomechanical testing is ongoing to determine the utility of the calibration device in aligning the COR of the TOPS construct with the COR of the ADR in cadaveric specimens. Results of this study will be published separately.

Future publications specific to the biomechanical testing are forthcoming.

The authors would like to apologise for any inconvenience caused.

